# An Epitope on EGFR Loading Catastrophic Internalization Serve as a Novel Oncotarget for Hepatocellular Carcinoma Therapy

**DOI:** 10.3390/cancers12020456

**Published:** 2020-02-16

**Authors:** Dianshuai Huang, Qingjie Fan, Zhiyi Liu, Shuqin Zhang, Wei Huang, Hongrui Li, Chongyang Liang, Fei Sun

**Affiliations:** 1 Institute of Frontier Medical Science, Jilin University, Changchun 130021, Jilin, China; huangds12@mails.jlu.edu.cn (D.H.); liuzq@jlu.edu.cn (Z.L.); sqzhang@jlu.edu.cn (S.Z.); huangw_jlu@jlu.edu.cn (W.H.); hongrui0924@jlu.edu.cn (H.L.); liang@jlu.edu.cn (C.L.); 2 Department of Biopharmacy, School of Pharmaceutical Sciences, Jilin University, Changchun 130021, Jilin, China; fanqj16@mails.jlu.edu.cn

**Keywords:** EGFR, HCC, internalization, epitope, target therapy

## Abstract

The precise role of Epidermal Growth Factor Receptor (EGFR) in Hepatocellular carcinoma (HCC) cells is unknown and EGFR inhibitors have not achieved positive clinical results. The rapid and drastic internalization of EGFR has been proved to successfully treat EGFR inhibitor-resistant patients in recent clinical trials. Here, the anti-tumor efficacy of a protein (rLZ-8) from *Ganoderma lucidum* was evaluated, it was demonstrated that rLZ-8 could bind to EGFR specifically, drastically enter into Hepatoma cells, abrogate endosomal recycling and induce HCC cell death. Surprisingly, we screened a monoclonal antibody which possesses competitive binding site with rLZ-8, it also trigger catastrophic EGFR internalization. This result suggests that it is necessary to investigate the interface of EGFR and rLZ-8 complex. An internalization related epitope (S222/K269) was identified on the dimerization arm of EGFR extracellular domain (ECD). These results suggest vulnerability of HCC cells to catastrophic EGFR internalization that can be targeted by a novel epitope and point to the possible exploitation in the design of anti-EGFR therapeutic biologics for HCC therapy.

## 1. Introduction

As a well-known therapeutic onco-target, epidermal growth factor receptor (EGFR) has played an important role in the treatment of various tumors, such as lung cancer [1]. Two main anti-EGFR strategies are currently exploited—anti-EGFR monoclonal antibodies, such as Cetuximab and Panitumumab, which bind directly to the ligand-binding site on the ectodomain, disturb the activation of downstream signaling pathways; tyrosine kinase inhibitors, such as Erlotinib and Gefitinib, that could internalize into cells and inhibit the phosphorylated activation by binding with the intracellular tyrosine kinase site of EGFR [2,3]. However, those anti-EGFR agents have not been proved positive evaluation in hepatocellular carcinoma (HCC) despite the expression of EGFR is commonly high [4,5,6]. Lin et al. reported that the anti-EGFR drug resistance in HCC is caused by the promoted interaction of EGFR with mTORC2, this shows that the complex signaling pathways is the main reason of drug resistance in liver cancer cells [7]. At present, the unique international recognized drug could benefit HCC patients is Sorafenib. Due to inhibiting multiple intracellular pathways, Sorafenib has several shortcomings such as insufferable side effects and short prolonging survival. Therefore, it still lacks more specific targeted drugs in HCC clinical therapy.

The internalization of EGFR is extremely complicated and most studies focus on the pathway and intensity of its internalization [8,9,10,11]. It has been reported that EGFR can enter cells by various endocytic pathways and the combinations of monoclonal antibodies induce more internalization of EGFR [12,13,14]. A synergistic antibody combination containing two antibodies which bind to different epitopes of EGFR, called as Sym004, induced rapid internalization and degradation of EGFR that leads to down-regulation of EGFR and subsequent inhibition of cancer cell growth [15,16]. In 2017, Sym004 was advanced through a Phase II trial in patients with metastatic colorectal cancer (mCRC) that have acquired resistance to anti-EGFR antibody therapies or mutations in RAS and BRAF and the data demonstrated that Sym004 could provide survival benefits or stabilization [17]. It can be seen that rapid internalization and recycling blockage of EGFR may be a novel strategy to develop anti-EGFR agents for HCC therapy.

A protein (Lingzhi-8, LZ-8) from *Ganoderma Lucidum* was reported that it had anti-tumor activity and could modulate EGFR expression but its binding site on EGFR and mechanism are still unclear [18,19]. Here, we found that recombinant LZ-8 (rLZ-8) could bind to EGFR specifically, induce catastrophic macropinocytosis, enter into HCC cells with EGFR, lead to the blockage of cell membrane recycling and then result in cell death via membrane ruffling, cell membrane over-internalization, cell rounding and bursting. Surprisingly, the new antibody which possesses competitive binding site with rLZ-8 also induced rapid internalization of EGFR in HCC cells. The above results caught our attention due to the possibility of the epitope as a novel oncotarget for HCC therapy. Then we analyzed the interface of rLZ-8 and EGFR complex, the binding site of EGFR was located on EGFR extracellular domain (ECD) Domain II and the key residues were S222/K269. In general, we present data from discovery process and functional characterization of an internalizing-epitope on EGFR. These findings highlight rapid internalization of EGFR as a promising strategy to maximize EGFR inhibition that may induce more potent HCC tumor suppression than current clinically anti-EGFR agents.

## 2. Results

### 2.1. Attenuation of Tumor Growth and Prolonging Survival Induced in Orthotopic HCC NOG-Mouse Model by rLZ-8

**Figure 1:**
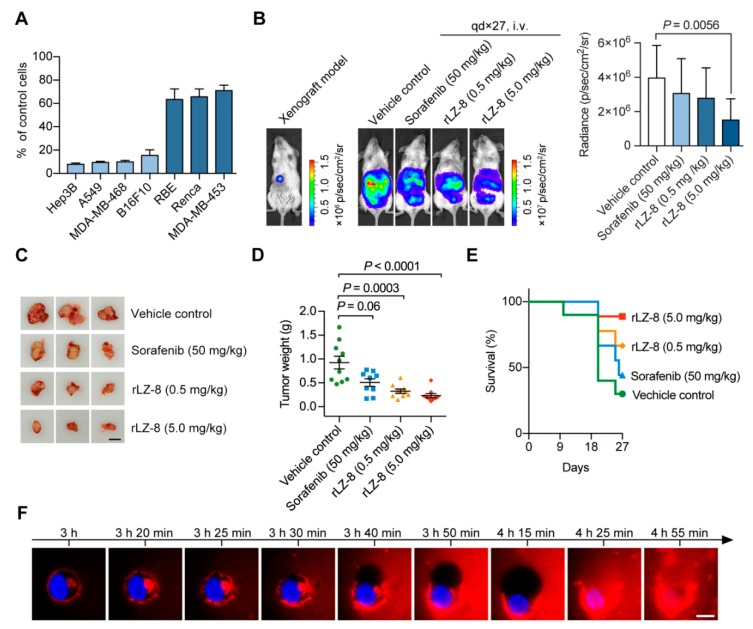
rLZ-8 could cause tumor cell death in vivo and in vitro. (**A**) Effect of rLZ-8 on the cell viability in vitro were detected after 48 h rLZ-8 incubating. (**B–E**) Hep3B cells were grown as orthotopic xenografts. Mice were divided into groups and dosed with normal saline control, 50 mg/kg Sorafenib or rLZ-8 with different concentrations. *n* = 9 per group. All mice were fed for 27 days. (**B**) Representative bioluminescence images obtained at day 0 or 27. (**C**) Images of HCC tumors dissected at 27 d post inoculation. All tumors of survival mice were imaged in every group. Bars, 1 cm. (**D**) Tumor weights were measured after dissection. (**E**) The survival status of mice was observed every day. (**F**) Hep3B cells real-time imaging began from 100 μg/mL rLZ-8 (red) treating for 3 h. Cell nuclei were dyed by Hoechst (blue). Representative images of death process were shown in this panel and the video of whole process was shown in Video S1. Scale bars, 10 μm. All data in A, B and D are means ± SD; two-tailed Mann-Whitney test. *p*-values as shown in panels.

We evaluated the anti-tumor activities of rLZ-8 in vitro and in vivo, respectively, with high purified rLZ-8 recombinant expressing in *Pichia pastoris* (Figure S1). In the cell viability assay in vitro, the growth of Hep3B, A549, MDA-MB-468 and B16F10 cancer cells was inhibited significantly (Figure 1A). In contrast, the growth of RBE, Renca and MDA-MB-453 cells was not interfered with. Here we selected Hep3B cell line to perform the anti-tumor test in immunodeficient NOG mice and selected Sorafenib as the positive drug control.

Orthotopic HCC NOG-mouse models were used to evaluate the anti-tumor efficacy of rLZ-8. Our results suggested that rLZ-8 inhibited the growth of tumor xenograft gradually with increasing doses (Figure 1B–E). The survival rate of the rLZ-8-treated group was improved significantly compared with vehicle control group (Figure 1E). Then the process of Hep3B cell death induced by rLZ-8 was observed via three-dimensional structured illumination microscopy (3D-SIM) live-cell imaging, the results showed that ruffling, contraction and rounding occurred consecutively in the cells (Figure 1F, Video S1). Finally, cell burst and cell death were observed, due to the lack of plasma membrane and inner space.

### 2.2. rLZ-8 Induces Catastrophic Macropinocytosis

**Figure 2:**
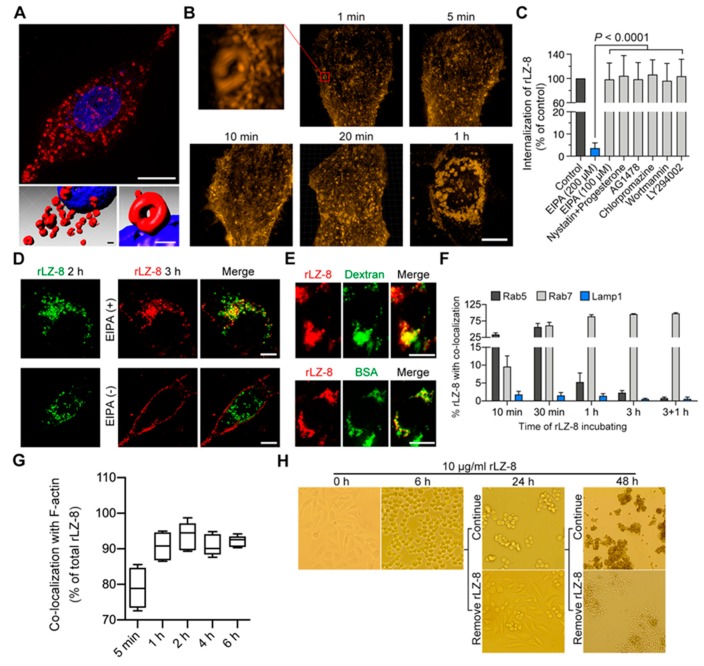
rLZ-8 induced intense macropinocytosis and blockage of endosomal recycling. (**A**) Ring-like vesicles appeared near the nuclei after 10 μg/mL rLZ-8 (red) treating on Hep3B cells for 1 h by 3D-SIM imaging. Cell nuclei were dyed by Hoechst (blue). Surface rendering was generated from Imaris. Scale bars in above, 10 μm, in below, 1 μm. (**B**) 10 μg/mL rLZ-8 (red) was treated on Hep3B cells for different time before cells imaging. Scale bars, 10 μm. (**C**) 5 μg/mL rLZ-8 treated on Hep3B cells for 2 h after different inhibitors pre-incubated for 30 min. Internalization of rLZ-8 was analyzed by Imaris. Statistical significance was calculated with a nonparametric Kruskal-Wallis test, in which a *p* < 0.0001 was considered significant. (**D**) 5 μg/mL rLZ-8 (green) treated on Hep3B cells for 2 h, then be removed. Same dose rLZ-8 (red) added after EIPA (200 μM) pre-incubated for 30 min. Cells were imaged 2 h later. Scale bars, 10 μm. (**E**) 10 μg/mL rLZ-8 (red) and 100 μg/mL Dextran or BSA (green) co-treated on Hep3B cells for 1 h before cells imaging. Scale bars, 10 μm. (**F**) Immunofluorescent staining of Rab5, Rab7 or LAMP1 after 10 μg/mL rLZ-8 treated for different time. For 3 + 1 h group, rLZ-8 was removed after treatment for 3 h and cells incubation lasted 1 h. The co-localization with rLZ-8 was analyzed by Imaris software. (**G**) Immunofluorescent staining of F-actin after 10 μg/mL rLZ-8 treated on Hep3B cells. Co-localization was analyzed by Imaris. (**H**) Live imaging of Hep3B cells using phase contrast microscopy. Three groups of cells were exposed to 10 μg/mL rLZ-8. In 2 groups, rLZ-8 treatment was discontinued after 6 h or 24 h of exposure. Magnification × 200 for 0 h, 6 h and 24 h imaging and × 40 for 48 h imaging. All data are means ± SD.

As illustrated in Figure 1F and Figure S2, a large amount of rLZ-8 could be intensely internalized into the cell. By using Imaris software to render super resolution live-cell imaging data, many vesicular organelles containing rLZ-8 were distributed around the nucleus. As shown in Figure 2A, after rLZ-8 was internalized, circular vesicle structures with a diameter of 0.5–2.0 µm was found in the perinuclear area. Therefore, it was deduced that rLZ-8 entered the tumor cells via encapsulation. 

Then we observed the whole process of rLZ-8 internalization. As shown in Figure 2B, after treating for 1 min, rLZ-8 had been abundant binding to cell membrane and the internalized cup-like structure had appeared on the membrane. With 5 min treating, the tubular endosomes began to form and increased over time. Mass endosomes accumulated around the nucleus in cytoplasm with 1 h rLZ-8 internalizing. Above all, rLZ-8 could induce rapid and catastrophic internalization in Hep3B cells.

To clarify how rLZ-8 enters the Hep3B cells, we used the inhibitors for clathrin-dependent pinocytosis, caveolae-dependent pinocytosis and macropinocytosis to investigate the rLZ-8 internalization mechanism, respectively (Figure 2C and Figure S3). EIPA, an inhibitor of macropinocytosis, inhibited rLZ-8 internalization significantly compared with other inhibitors (Figure 2D). It is noteworthy that we used twice usual dose of EIPA (200 μM) to inhibit the internalization of rLZ-8, with the general dose (100 μM) not interfering, which is further confirmed the high strength of rLZ-8 internalization.

As shown in Figure 2E, the marker of macropinocytosis (dextran) and the internalized substance of macropinocytosis (bovine serum albumin, BSA) were both co-localized with rLZ-8 distinctly in the cells. Taken together, rLZ-8 could internalize into tumor cells by catastrophic macropinocytosis, which may be particular and remarkable. (Added descriptions of macropinocytosis characterization were shown in Supplementary Material 1 and Figure S4).

### 2.3. Endosomal Recycling Is Abrogated by Continuous Internalization of rLZ-8

Here, we further explored more details in the internalization of rLZ-8. Based on the above results, the role of early endosomes (EE), late endosomes (LE) and lysosomes, the relative activity and cellular imaging were used in investigating the degradation of rLZ-8 internalization. As shown in Figure 2F and Figure S5, rLZ-8 and Rab5 (early endosome marker) co-localized after 10 min. After 30 min, the co-localization gradually disappeared, followed by co-localization of rLZ-8 and Rab7 (late endosome marker), which was maintained. During the internalization, rLZ-8 was not co-localized with Lamp1 (lysosome marker). These results indicated that the internalization of rLZ-8 stayed in the late endosome stage and was not fused with lysosomes, which led to abundant rLZ-8 accumulation. (Added descriptions of Rab7 activation and CaCl_2_ influence were shown in Supplementary Material 2 and Figure S6)

Notably, the effect of continuous accumulation of LE on the whole plasma membrane (PM) transport was investigated by imaging the PM recycling during rLZ-8 internalization. With the internalization of rLZ-8 at 5 min, 1 h, 2 h, 4 h and 6 h, rLZ-8 maintained highly co-localized with F-actin fragment, which was formation mediated via macropinocytosis on cell membrane (Figure 2G and Figure S7). Therefore, with rLZ-8 internalization, PM containing rLZ-8 did not recycle to the surface of cell membrane. As shown in Figure 2H, Hep3B cells were recovered to normal status at 24 h treatment with rLZ-8 being removed at 6h, by contrast, the cells died mostly with rLZ-8 24 h continuous treatment. The similar results were observed at 48 h group. Membrane ruffling and cell rounding were also observed with rLZ-8 internalization (Figure S8). Besides, rLZ-8 could abrogate the fusion between LE containing BSA and lysosomes (Figure S9). Above all, rLZ-8 resulted in the blockade of PM recycling by increased internalization of macropinocytosis, which might be attributed to cell shrinkage and death.

### 2.4. EGFR Is the Primary Receptor of rLZ-8 in HCC Cells

**Figure 3:**
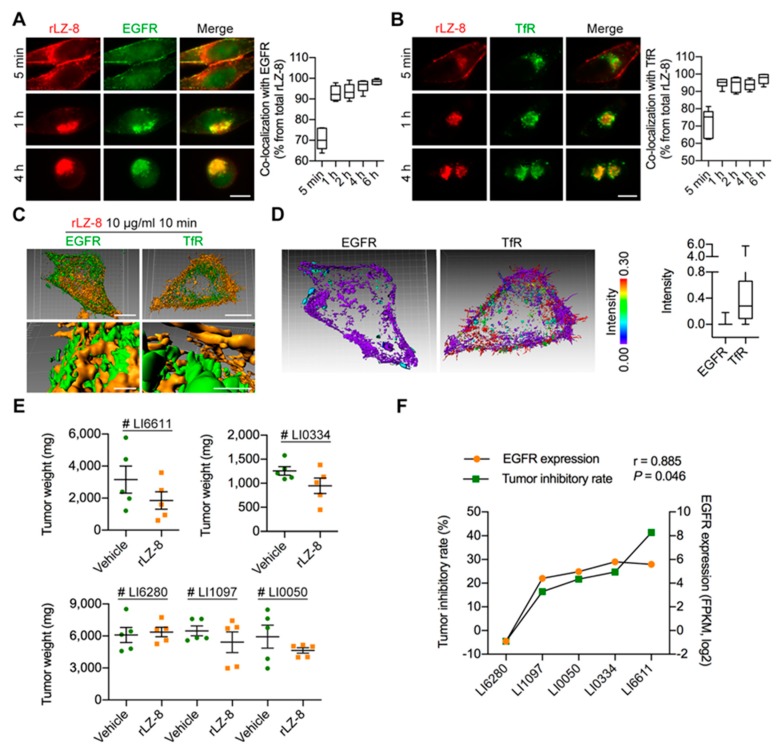
Epidermal growth factor receptor (EGFR) was the primary onco-target of rLZ-8. (**A,B**) Immunofluorescent staining of EGFR or trasnferrin (TfR) after 10 μg/mL rLZ-8 treated on Hep3B cells. Co-localization was analyzed by Imaris. Scale bars, 10 μm. (**C**) Hep3B cells were treated with rLZ-8, with EGFR/TfR coloring by immunofluorescence (no permeabilized). Surface rendering was generated from Imaris. Scale bars in above, 10 μm; in below, 1 μm. (**D**) Distance from rLZ-8 to the nearest EGFR/TfR was measured by Imaris. The varying distance transformed the intensity of fluorescence. (**E**,**F**) Different types of PDX models (LI6280, LI1097, LI0050, LI0334, LI6611) of HCC were established as shown in Materials and Methods. Mice were divided into groups and dosed with normal saline control or 5 mg/kg rLZ-8. All mice were injected once daily for total 28 days. (**E**) Tumors were dissected at 28 d post inoculation and imaged. Tumor weights were measured after dissection. (**F**) The correlation analysis between EGFR expression and tumor inhibitory rate. Pearson correlation coefficients. *p*-value and r-value as shown in panel. All data are means ± SD.

As illustrated in above results, the internalization of rLZ-8 maintained a constantly high internalization rate, which was higher than normal macropinocytosis, the results might be attributed to the internalization triggered by the interaction between rLZ-8 and plasma membrane, allowing some kind of receptor entry into the cells. In order to reveal this phenomenon, the co-localization of PM-specific receptors with rLZ-8 were observed, such as EGFR, transferrin (TfR), N-cadherin, c-Met, low-density lipoprotein receptor (LDLR) and platelet-derived growth factor receptor (PDGFR), which were closely related to macropinocytosis. As shown in Figure 3A,B, rLZ-8 was highly co-localized with EGFR and TfR at all times. In Figure 3C, EGFR and rLZ-8 seems to fuse as a whole shape but TfR and rLZ-8 displays the varied distance. Further we analyzed the distance between rLZ-8 and two receptors, only purple can be seen in the scale analysis of EGFR and rLZ-8, suggesting that rLZ-8 may bind with EGFR directly (Figure 3D). Conversely, rLZ-8 displayed no significant co-localization with N-cadherin, c-Met, LDLR and PDGFR (Figure S10). Thus, we concluded that EGFR is the primary receptor of rLZ-8 and the internalization rate of rLZ-8 may be related with EGFR.

In the cell viability assay in vitro, the growth of Hep3B, MDA-MB-468, A549 and B16F10 cells was inhibited significantly, all of cell lines was abnormally high EGFR expression (Figure 1A). However, similar results of efficacy were not observed in EGFR negative cell lines, RBE, Renca and MDA-MB-453. Then, we established five patient-derived tumor xenograft (PDX) models of HCC (LI6280, LI1097, LI0050, LI0334, LI6611) which have different expression of EGFR. As shown in Figure 3E and Figure S11, the effect of rLZ-8 on the attenuation of tumor growth was diversity among different models. The tumor inhibitory rate of rLZ-8 was correlative with the EGFR expression (Figure 3F and Figure S12).

**Figure 4:**
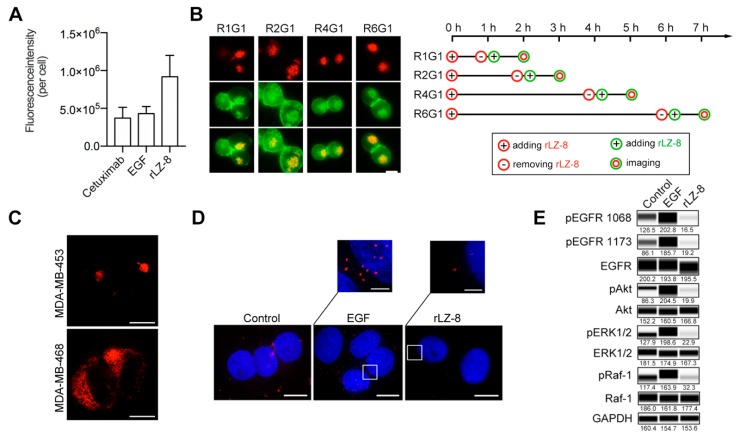
rLZ-8 was bound with EGFR by a specific binding interface. (**A**) 20 nM EGF, Cetuximab and rLZ-8 at same dose treated on Hep3B cells for 30 min, respectively. The internalization of epidermal growth factor (EGF), Cetuximab and rLZ-8 (fluorescence intensity per cell) were analyzed by Imaris software. All data are means ± SD. (**B**) 10 μg/mL rLZ-8 (red) treated on Hep3B cells for different time and then be removed. Then 10 μg/mL rLZ-8 (green) treated on cells for 1h before imaging. The order of adding rLZ-8 at varying levels are shown beside the panel. Scale bars, 10 μm. (**C**) 10 μg/mL rLZ-8 treated on MDA-MB-453 and MDA-MB-468 cells for 3 h before imaging. Scale bars, 10 μm. (**D**) In situ proximity ligation assay (PLA) detection (red) of EGFR homo-dimerization on Hep3B cells, stimulated with EGF (100 ng/mL)/rLZ-8 (10 μg/mL) for 3 min. Cell nuclei were counterstained with Hoechst (blue). Scale bars, 10 μm. (**E**) Hep3B cells were treated with rLZ-8 (10 μg/mL), EGF (100 ng/mL) for 15 min. The expression of relative proteins of EGFR pathways was detected using western blotting analysis.

Then the internalization rates of EGFR which induced by rLZ-8, epidermal growth factor (EGF) and an anti-EGFR antibody Cetuximab were compared together. In the same doses, EGFR which rLZ-8 induced was several times more than that EGF or Cetuximab (Figure 4A). And the rate of rLZ-8 internalization was not reduced obviously as continuous macropinocytosis (Figure 4B). Therefore, the high internalization rate of EGFR induced by rLZ-8 was particular and extraordinary.

Next the relationship between EGFR and rLZ-8 was further supported. As illustrated in Figure 4C, the entry of rLZ-8 into MDA-MB-453 cells (with low EGFR expression) were significantly lower than in MDA-MB-468 cells (with high EGFR expression) with the same concentrations. EGF binding is followed by dimerization of EGFR, phosphorylation and alteration of the downstream signaling pathway. Therefore, we firstly used the proximity ligation detection (PLA) technique to detect the dimerization status of EGFR after the rLZ-8 pre-treatment. As shown in Figure 4D, rLZ-8 binding failed to induce the dimerization of EGFR. In addition, western blot (Figure 4E and Figure S19) revealed that the EGFR phosphorylation level was not significantly changed after the rLZ-8 reaction and also the phosphorylation did not occur. Above all, rLZ-8 bound with EGFR on cell membrane and did not induce the dimerization and phosphorylation of EGFR, which meant that the binding form of rLZ-8 and EGFR may be specific. (Added descriptions of immunohistochemistry and siRNA were shown in Supplementary Material 3 and Figures S13 and S14)

### 2.5. The Anti-EGFR Antibody Which Possesses Competitive Binding Site with rLZ-8 Could Induce Catastrophic Internalization

**Figure 5:**
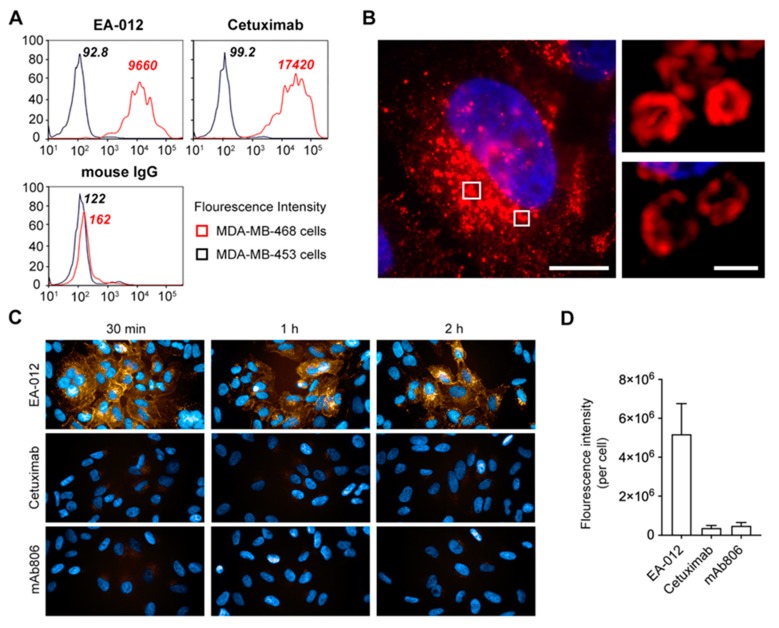
EA-012 could induce catastrophic internalization. (**A**) MDA-MB-468 cells was used as EGFR positive cells, with MDA-MB-453 as negative cells. Cells were stained with a negative control antibody (mouse IgG), a positive control antibody (Cetuximab) and EA-012. Then cells were analyzed by fluorescence activated cell sorting (FACS). (**B**) 10 μg/mL EA-012 was treated on Hep3B cells. Cell nuclei were dyed by Hoechst (blue). Surface rendering was generated from Imaris. Scale bars in left, 10 μm, in right, 1 μm. (**C,D**) EA-012, Cetuximab or mAb806 (red) treated on Hep3B cells for different times and the cells were imaged by high content imaging system. Cell nuclei were counterstained with Hoechst (blue). Magnification ×60. The internalization of EA-012, Cetuximab or mAb806 was analyzed by Harmony software. All data are means ± SD.

The human EGFR ECD was used as antigen to immunize BALB/c mice for antibody production. More than 300 clones were obtained with confirming to specifically bind to EGFR by ELISA. rLZ-8 was used to competitive screening, 3 subclones were confirmed and expressed for the following experiments. To identify the characteristic of new antibodies, we chose MBA-MD-453 as EGFR negative cells, MBA-MD-468 as EGFR positive cells and Cetuximab as positive antibody to detect the binding ability with EGFR by fluorescence activated cell sorting (FACS). There was most clone 3 (EA-012) binding on MBA-MD-468 cells (Figure 5A). Thus, we obtained a new anti-EGFR antibody (EA-012) which possesses competitive epitope with rLZ-8.

Then we evaluated the internalization of EA-012 in Hep3B cells. There was abundant EA-012 internalized into cells intensely and many endosomes containing EA-012 were distributed around the nucleus (Figure 5B). This is a surprising result that the internalized quantity of EA-012 was ten times more than mAb806 and Cetuximab at least (Figure 5C,D). In the cell viability assay in vitro, EA-012 did not show anti-tumor activity. Considering antibody screening is a long-term and complicated process, the antibodies both with rapid internalization and anti-tumor activity may need to be further developed. These results demonstrated that the anti-EGFR antibody which possesses competitive binding site with rLZ-8 could induce catastrophic internalization and the epitope on EGFR seems to have the potential to further development for EGFR targeted therapy.

### 2.6. Mapping the EGFR/rLZ-8 Binding Interface Using Chemical Cross-Linking Coupled with Mass Spectrometry (CXMS) and Molecular Docking

**Figure 6:**
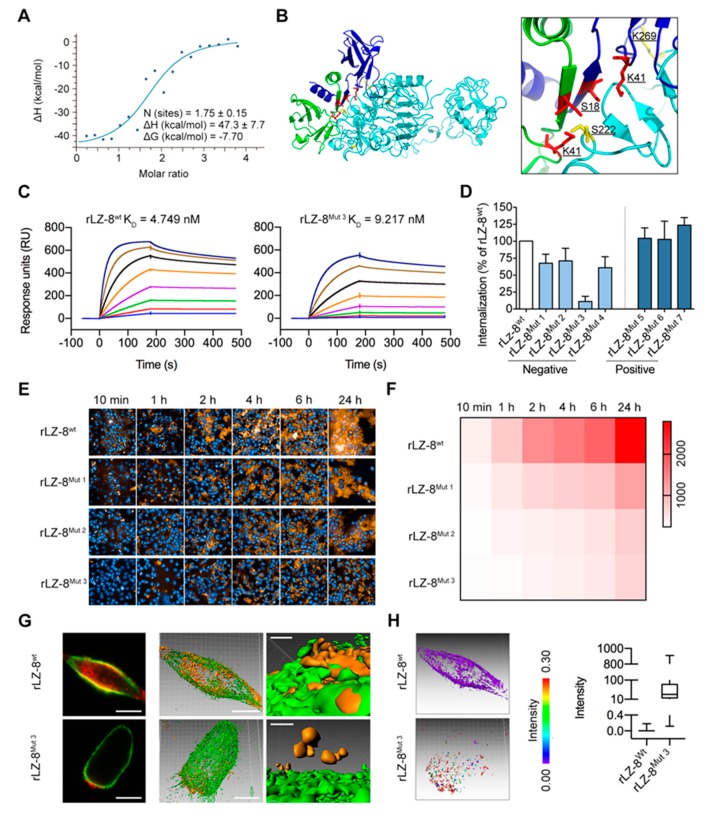
rLZ-8 strongly bound with EGFR via the key residue K41. (**A**) ITC experiments employing purified EGFR ectodomain and rLZ-8. The molar ratio was shown in panel. (**B**) Ribbon representation of identified interface of rLZ-8/EGFR complex, based on the results of chemical Cross-Linking Coupled with Mass Spectrometry (CXMS) and molecular docking. (**C**) Biacore analysis. Recombinant human EGFR was immobilized onto sensor chips and rLZ-8*^wt^* or rLZ-8*^Mut 3^* were injected at concentrations ranging from 4 nM to 512 nM. K_D_ values were shown on panels. (**D**) Hep3B cells were treated with 10 μg/mL rLZ-8*^wt^* or rLZ-8*^Mut 1-7^* for 1 h before cells imaging. The internalization of rLZ-8*^Mut 1-7^* was analyzed by Imaris. (**E,F**) rLZ-8*^wt^* or rLZ-8*^Mut 1-3^* (red) treated on Hep3B cells for different times and the cells were imaged by high content imaging system. Cell nuclei were counterstained with Hoechst (blue). Magnification ×60. The internalization of rLZ-8*^wt^* or rLZ-8*^Mut 1-3^* was analyzed by Harmony software and the thermal figure was shown in (**F**). (**G**) Hep3B cells were treated with 10 μg/mL rLZ-8*^wt^* or rLZ-8*^Mut 3^* (red) for 20 min, with EGFR (green) coloring by immunofluorescence (no permeabilized). Surface rendering was generated using Imaris. Scale bars in left 2 columns, 10 μm; bars in right 1 columns, 2 μm. (**H**) Distance from rLZ-8*^wt^*/rLZ-8*^Mut 3^* to the nearest EGFR was measured by Imaris. Varying distances modify fluorescence intensity. All data are means ± SD.

In order to investigate the molecular details of intense internalization of rLZ-8 and EGFR, CXMS and molecular docking of rLZ-8 and EGFR was performed. To characterize the stoichiometry of the EGFR/rLZ-8 complex, we conducted isothermal titration calorimetry (ITC) experiments employing purified EGFR ECD and rLZ-8. It was demonstrated that EGFR ECD binds to rLZ-8 in the molar ratio of 1:2 (Figure 6A and Figure S15A), indicating that one molecule of EGFR binds to a rLZ-8 dimer. 

To map the interface of the EGFR/rLZ-8 complex, the purified EGFR ECD and rLZ-8 proteins were subjected to CXMS studies, which could provide distance information by identifying lysine and serine residues that are proximate to each other and have been covalently bound via the bifunctional cross-linking reagent disuccinimidyl suberate (DSS), or bissulfosuccinimidyl suberate (BS3), the water-soluble version of DSS. Due to DSS/BS3 molecules impose ~30 Å Cα distance constraints on the corresponding proximal lysine residues, this technique could allow us to obtain the low-resolution information on the 3D structures of protein complexes. The cross-linked 1:2 EGFR/rLZ-8 complexes with DSS/BS3 were isolated by SDS-PAGE (Figure S15B) and digested by trypsin and subjected to tandem MS analysis.

In total 26 cross-linked peptides were identified, including three high-confidence intermolecular cross-linked peptides which describes the EGFR/rLZ-8 protein interface. The resulting BS3/DSS cross-linked sites were mapped onto both the crystal structure rLZ-8 and EGFR ECD. The identified interface of the EGFR/rLZ-8 complex involved K41 from the loopBC of rLZ-8 and S196, S222 and K269 from the dimerization arm (DA) of EGFR ECD. We also performed zero-length cross-linking of EGFR/rLZ-8 complex using N-Hydroxysuccinimide/1-ethyl-3-(3-dimethylaminopropyl) carbodiimide hydrochloride (NHS/EDC) to validate the BS3/DSS cross-linking data, and one unique inter-chain cross-links involving D20 of rLZ-8 and S282 of EGFR ECD was identified.

In order to further investigate the molecular details of intense internalization of rLZ-8 and EGFR, molecular docking of rLZ-8 and EGFR was performed, we found that the two residues of EGFR (S222 and K269) interacting with rLZ-8 (S18 and K41) were a key region for catastrophic internalization (Figure 6B). In general, the identified interaction regions between EGFR ECD and rLZ-8 suggest that rLZ-8 could directly block the dimerization interface to prevent the formation of functional EGFR dimer in the presence of EGF, thus inhibiting the EGFR signaling.

### 2.7. Targeting S222/K269 Switches Catastrophic EGFR Internalization by Key Residue K41 of rLZ-8

To further verify more details of the interaction between rLZ-8 and EGFR, we designed and expressed seven rLZ-8 mutants (Table 1). Then we compared the internalization rate of each mutant and their affinity with EGFR and evaluated the relationship between the previously mentioned amino acid residues of rLZ-8 and its the internalization ability. Biacore assay demonstrated that the affinity of rLZ-8*^Mut 1-4^* with EGFR decreased to varying degrees compared to the wild-type rLZ-8, with a significant decrease (more than 50%) in the affinity of rLZ-8*^Mut 3^* (Figure 6C). Other mutants did not show significant changes (Figure S16), suggesting that the domain centered on K41 may play an important role in the recognition of EGFR.

The data in Figure 6D–F showed that internalization of rLZ-8*^Mut 1-4^* in Hep3B cells reduced significantly comparing with that of rLZ-8*^wt^* and that was consistent with the previous results of Biacore assay, while no significant changes were observed in internalization assay of other mutants. By further evaluating the binding rate of rLZ-8*^Mut 1-4^* and EGFR, only a few rLZ-8*^Mut 3^* bound to the cell membrane (Figure 6G) but Imaris analysis showed a clear distance between rLZ-8*^Mut 3^* and EGFR positions (Figure 6H). Taken together, K41 in rLZ-8 crystal structure is a key residue in binding with EGFR. (Added descriptions of binding analysis were shown in Supplementary Material 4 and Figures S17 and S18)

**Figure 7:**
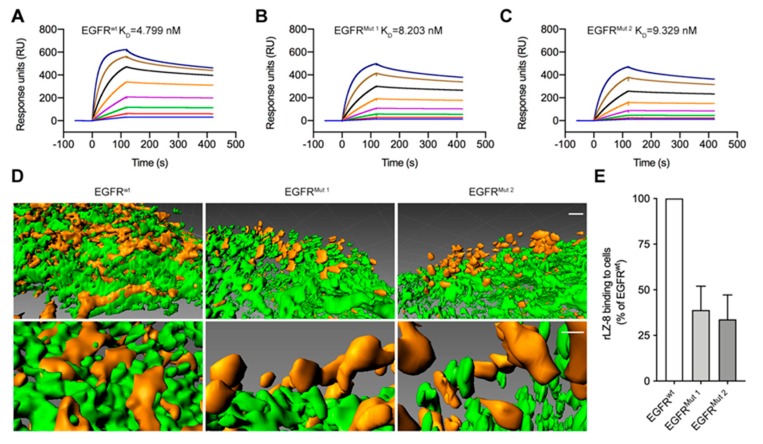
(S222, K269) was the binding sites of EGFR related to intense internalization. (**A–C**) Biacore analysis. rLZ-8 was immobilized onto sensor chips and EGFR*^wt^* EGFR*^Mut 1^* or EGFR*^Mut 2^* were injected at concentrations ranging from 4 nM to 512 nM. K_D_ values were shown on panels. (**D**) 10 μg/mL rLZ-8 (red) was treated on NIH-3T3-EGFR*^wt^*^/*Mut 1*/*Mut 2*^ cells, with EGFR (green) coloring by immunofluorescence. Surface rendering was generated from Imaris. Scale bars above, 2 μm, bottom, 1 μm. (**E**) The binding quantity of rLZ-8 to cells was analyzed by Imaris software. All data are means ± SD.

To investigate the key amino acid residues in EGFR structure bind to rLZ-8, a new target for catastrophic internalization was explored. Based on the results of the previous CXMS and molecular docking, we designed two EGFR ECD mutants (Table 1) and tested their affinities with wild-type rLZ-8 and its mutants (Figure 7A–C). Comparing wild-type EGFR with EGFR*^Mut 1-2^*, both have a lower ability to bind to rLZ-8, suggesting that S222 and K269 of EGFR play an important role in the binding to rLZ-8. On this basis, two residues (S222 and K269) or four residues (S196, S222, K269 and S282) were replaced in EGFR ECD with alanine, and expressed EGFR*^wt^*, EGFR*^Mut 1^* or EGFR*^Mut 2^* in NIH-3T3 cells. The binding of rLZ-8 with EGFR in these cell lines was investigated, as shown in Figure 7D,E, there was significantly less rLZ-8 binding with EGFR on NIH-3T3-EGFR*^Mut 1^* cells and NIH-3T3-EGFR*^Mut 2^* cells than that on NIH-3T3-EGFR*^wt^* cells. Therefore, S222 and K269 in EGFR are the binding sites related to the internalization activity of rLZ-8, which could be developed to be a novel oncotarget for HCC therapy.

## 3. Discussion

Hepatocellular carcinoma (HCC) is an aggressive tumor and typically diagnosed late in the course of the chronic liver disease [20,21]. Due to the high rate of expression of drug resistance genes, the degree of hepatic dysfunction or attendant cirrhosis frequently, there is few effective therapy existed for patients with advanced-stage HCC [22]. At present, Sorafenib (a multitargeted small molecule tyrosine kinase inhibitor (TKI)) is the unique first-line therapy drug of advanced HCC which is approved by the Food and Drug Administration (FDA) [23,24,25,26]. In the second-line therapy, Nivolumab (a PD-1 antibody) and Regorafenib (a vascular endothelial growth factor receptor (VEGFR) inhibitor) have been preliminary used, respectively or combined, especially in patients who have been previously treated with Sorafenib [27,28]. Besides, another VEGFR/fibroblast growth factor receptor (FGFR)/PDGFR inhibitor Lenvatinib, which has not merely been approved by FDA, is a reasonable first-line alternative to Sorafenib, especially for patients who cannot tolerate Sorafenib [29,30]. Therefore, all existing strategies are difficult to be efficient applied, including blocking the binding between receptors and ligands by monoclonal antibodies, inhibiting the intracellular amino acid kinase sites by small molecule inhibitors and checkpoint inhibitor immunotherapy, it is obvious that further development of available mechanisms cannot receive any more breakthrough achievement. In consideration of perspective and applicability, finding a new anti-tumor mechanism in HCC clinical therapy is essential.

There have several reported that the epidermal growth factor receptor (EGFR) pathway plays an important role in the carcinogenesis and proliferative behavior of HCC [31,32,33,34]. These lead to the exploration of anti-EGFR agents in HCC therapy and four anti-EGFR strategies have been probed: small molecule TKIs (Erlotinib), Erlotinib plus Bevacizumab, Erlotinib plus Sorafenib and Cetuximab [35,36,37,38,39,40]. However, the above strategies have rarely efficient antitumor activity in HCC and the reasons are complicated, including promoted interaction of EGFR with mTORC2, cross-talk between EGFR families and the complex signaling pathways [7]. In our studies, rLZ-8 bound with EGFR but did not cause dimerization of EGFR and activation of its signaling pathways, it caused catastrophic internalization of EGFR and blockade of membrane recycling, with consequent cell shrinkage and death. In other words, the binding site (S222/K269) of rLZ-8 in EGFR is located on Domain II of EGFR extracellular domain (ECD) and therefore did not disturb the cell signaling pathways which was different with Sorafenib. As most marketed anti-EGFR mAbs are targeted on Domain I/III of EGFR ECD, this may explain why the epitope has anti-tumor activity in HCC, distinguishing it from anti-EGFR antibodies and TKIs.

Our studies indicated that the rate and intensity of rLZ-8 internalization were much higher than of EGF or monoclonal antibodies. Surprisingly, similar rapid internalization was observed in Hep3B cells after the new antibody with similar epitope on EGFR incubating. The present study raises the possibility that the antibody with this epitope could induce rapid internalization and be developed as new anti-EGFR agent. After internalization of rLZ-8, abundant late endosomes formed after the combination of rLZ-8 and EGFR could not fuse with the lysosomes and degraded. So plasma membranes on LE did not recycle to the surface of cell membrane. However, the reasons about high intensity of rLZ-8 internalization and non-recycling to the cell surface of plasma membranes still confused us, in future we will focus these processes to find unknown mechanisms.

In conclusion, the epitope in EGFR that we found exhibited specific functional characterization. We deduced that the epitope could cause catastrophic internalization of EGFR via macropinocytosis, recycling blockage of cell membrane, cell membrane over-internalization, cell rounding and bursting, which have the potential to be a new anti-EGFR mechanism for HCC clinical therapy. In future investigations, more antibodies should be developed to further verify the novel oncotarget.

## 4. Materials and Methods

### 4.1. Recombinant Plasmid Construction and Pichia Pastoris Transformation

The codons of wild-type and mutant LZ-8 genes were optimized according to the Codon Adaptation Tool (http://www.jcat.de) [41]. To clone the gene into the pGAPZα A vector (Thermo Fisher Scientific, Waltham, MA, USA), restriction sites Xho I and Xba I were introduced at the 5’- and 3’- ends of the optimized sequence, respectively. The optimized genes were synthesized by GenScript (Nanjing, China) and cloned into pGAPZα A vectors. The recombinant wild-type and mutant LZ-8 proteins were expressed in *Pichia pastoris* X33 (Mut^+^, Thermo Fisher Scientific) and the transformation according to the manufacturer’s instructions.

### 4.2. Media and Culture Conditions for rLZ-8 Expression

The *P. pastoris* transformants were cultured in a 2 L flask containing 400 mL YPD medium, supplemented with 1% (*v/v*) glycerol as a carbon source and 200 μg/mL G418 (Geneticin) as a selection pressure. Cells were grown at 28.5 °C and shaken at 225 rpm until an OD600 value of 6 had been reached and the cells were added to a BioFlo 310 Bioreactor (New Brunswick scientific, Enfield, CT, USA), which had been filled with 3.5 BSM medium, 8 mL Biotin and 12 mL PTM1. The culture conditions were 800 rpm, 29 °C and 20% dissolved oxygen. Glycerol was supplemented constantly to ensure rLZ-8 expression. Samples were taken every 6 h and the rLZ-8 expression was detected by SDS-PAGE (Figure S1A). After 96 h of induction, the supernatant was collected by centrifugation at 4 °C and 12,000 g for 10 min. rLZ-8 protein was purified by a Superdex TM-75 prep grade column (GE Healthcare, Pittsburgh, PA, USA) and eluted by a gradient of 30–100 mM imidazole. The purity of rLZ-8 reached 99.661%, analyzed by HPLC (Figure S1B).

### 4.3. Cell Lines

The human hepatocellular cancer cell line Hep3B, human breast carcinoma cell line MDA-MB-453 and MDA-MB-468, mouse hepatoma cell line H22, human lung carcinoma cell line A549, mouse melanoma cell line B16-F10, human cholangiocarcinoma cell line RBE, mouse renal carcinoma cell line Renca, primary aortic endothelial (PAE) cells and NIH-3T3 cells were purchased from the American Type Culture Collection. NIN-3T3 cells expressing wild-type or mutant EGFR (S222A/K269A or S196A/S222A/K269A/S282A) were generated as described previously [42].

### 4.4. Cell Viability Assays

CytoTox-FluorTM Cytotoxicity Assay (Promega Corporation, Madison, WI, USA) was used to quantify cell viability. The assay uses a fluorogenic peptide substrate (bis-AAF-R110) to measure “dead-cell protease activity,” which has been released from cells that have lost membrane integrity. The bis-AAF-R110 Substrate cannot cross the intact membrane of live cells and therefore gives no signal from live cells. The viability assays were performed following the manufacturer protocol.

### 4.5. Xenograft Mouse Model In Vivo

Orthotopic xenografts were developed using six-week-old male BALB/c mice housed in individual ventilated cages. Hep3B cells were grown in mouse liver. After the tumor volumes reached 800–1000 mm^3^, 50 mg/kg Sorafenib, rLZ-8 at different concentrations or normal saline control were administered to the mice by intravenous injection (i.v.). Mice were injected once daily (qd) or once every 3 days (q3d) for 27 days. The tumors were dissected and imaged. Bioluminescence images were obtained daily using IVIS Spectrum In Vivo Imaging System from PerkinElmer (Waltham, MA, USA).

PDX models were established in Crown Bioscience (Taicang, China) using tumor fragments, which were subcutaneously transplanted and passaged in male BALB/c nude mice (Beijing HFK Bioscience Co. Ltd., Beijing, China). All the animal experiments were performed in Crown Bioscience under sterile condition in an SPF facility and conducted in accordance with the animal welfare laws and the regulations of the Association for Assessment and Accreditation of Laboratory Animal Care (AAALAC). The BALB/c nude mice were housed in individual ventilated cages and used at 6 weeks of age. HuPrime^®^ liver cancer xenograft models (LI6280, LI0334, LI1097, LI0050 and LI6611) and subcutaneous tumors were revived and maintained in BALB/c nude mice, before orthotopic implantation. When the tumor volumes reached 500–1000 mm^3^, tumors were collected and fragmented into pieces measuring about 8 mm^3^ in diameter each and inoculated into the left lobe of the liver in nude mice. The day of implantation was designated as day 0 and 5 mg/kg rLZ-8 or normal saline controls were administered to mice intravenously (i.v.). All the mice were injected once daily (qd) for 28 days. Tumors were dissected after a 28-d inoculation and measured or imaged.

### 4.6. Immunofluorescence, Microscopy Imaging and Analysis

Immunofluorescence was accomplished by growing cells on glass coverslips and adding rLZ-8, EGF or other reagents at the time-points that we set. The cells were fixed with 4% paraformaldehyde in phosphate-buffered saline (PBS) for 20 min and permeabilized with 0.1% Triton X-100 for 10 min at room temperature, followed by incubation with primary antibody for 1 h at room temperature and washed (3 times, 5 min each) with PBS/serum before incubation with relevant secondary Alexa Fluor antibodies at 1:1000 for 1h. Live cell imaging was performed in 20 mm dishes following the addition of rLZ-8 or other reagents. Cell nuclei were dyed by Hoechst 33342.

Cells were imaged using a DeltaVision OMX Imaging System with 3D-SIM model provided by GE Healthcare (No.OM06051) and a High Content Operetta CLS Imaging System from PerkinElmer. The channel, mode, exposure, excitation and luminescence were regulated for optimum imaging. The softWoRx (GE Healthcare) was used to reconstruct the image data from OMX. Volume rendering of the reconstructed image was also accomplished using softWoRx. Image rendering and analysis was carried out with Imaris software from Bitplane (Zurich, Switzerland). Surface and spot modules were used to color different imaging channels. Region of interest (ROI) imaging provided the surface position information. The slice view measured and analyzed the relative data between imaging structures.

Transmission electron microscopy (TEM) was carried out on a Tecnai F30 high-resolution TEM from FEI Company (Hillsboro, OR, USA). The samples were prepared and TEM performed essentially as described previously [43].

### 4.7. Chemical Inhibitors

Hep3B cells were incubated on 20 mm dishes. Before addition of rLZ-8, cells were pre-treated with different inhibitors for 30 min. The inhibitor concentrations were as follows: EIPA 100 μM or 200 μM, Wortmannin 100 nM, AG1478 1 μM, Chlorpromazine 25 μM, LY294002 50 μM, Nystatin 25 μg/mL, Progesterone 10 μg/mL and mβCD 1 mM.

### 4.8. Isothermal Titration Calorimetry

Calorimetric binding experiments were performed using a MicroCal VP-ITC instrument (GE Healthcare). Nineteen successive 3 μL aliquots of 400 μM rLZ-8 were injected into a sample compartment containing 20 μM EGFR ectodomain. ITC data were analyzed via the MicroCal Origin software program employing a single site binding model and nonlinear least squares analysis of ΔH, K and n to derive the requisite thermodynamic binding parameters.

### 4.9. Chemical Cross-Linking Coupled with Mass Spectrometry (CXMS) and Molecular Docking

Cross-linking reactions were performed as described previously [44]. Briefly, the EGFR ectodomain (25 μM) and rLZ-8 (50 μM) were incubated in 20 mM phosphate buffer, 150 mM NaCl (pH 7.4) with DSS/BS3 in a 1:4 ratio for 1 h at room temperature and the cross-linking reaction was quenched with 50 mm Tris (pH 7.4). The DSS/BS3 and zero-length cross-linked complexes were separated on SDS-PAGE gels, stained with Coomassie Blue and the 1:2 EGFR-rLZ8 complexes were excised and stored in 10% acetic acid for mass spectrometry (MS) analysis.

Reduction and alkylation of cysteine residues were performed using Tris-(carboxyethyl) phosphine hydrochloride (TCEP) and chloroacetamide (CAA) for 60 min at 56 °C and for 45 min at room temperature, respectively. Each sample was digested with trypsin at 37 °C overnight and the generated peptides were separated on a nano-LC system (Easy-nLC 1200, Thermo Fisher Scientific) operating in a reverse-phase mode at a flow rate of 300 nL/min. Nano-LC solvents were A (0.1% formic acid in water) and B (95% CAN and 0.1% formic acid in water). The system was connected to a nano-ESI source coupled to Orbitrap Fusion Lumos mass spectrometer (Thermo Fisher Scientific). Mass spectrometry data were acquired continuously over the whole gradient. Each MS scan was acquired in the orbitrap over a mass range of m/z 400–2000 at a resolution of 70,000 (m/z 400), followed by the 10 data-dependent acquisition mode controlled by XCalibur 2.0 software (Thermo Fisher Scientific). The 10 most intense signals in the mass spectrum were selected for higher-energy collision dissociation and fragments were detected by Orbitrap at a resolution of 35,000 (m/z 400). Cross-linked peptides were identified using the SIM-XL software program followed by manual validation.

Based on the crystal structure of rLZ-8, we developed a model of mPEG-SPA-rLZ-8. The molecular docking was performed using AMBER 12 software package. The force-field is AMBER99SB (ff99SB) force field [45]. The sodium ions (Na^+^) or chloride ions (Cl^−^) were added by the t-Leap to be the explicit net neutralizing counterions based on a coulomb potential grid. For those complexes that were further subjected to molecular dynamics (MD) simulations in explicit solvent, each system was solvated with TIP3P waters in a truncated octahedron box, with a 10.0 Å distance around the solute. 50 ns simulation for each system under NPT ensemble condition was performed [46]. Then, PEG16 was docked into the active site of the protein using the CDOCKER protocol of Discovery Studio 2.5 (Dassault Systemes BIOVIA, Velizy-Villacoublay, France). Based on the results of MD simulation and docking, mutation schemes were designed. All the amino acids that may be affected were mutated into ALA using Discovery Studio 2.5. The electrostatic potential analysis was calculated using PyMOL software (Delano Scientific, Palo Alto, CA, USA).

### 4.10. Determination of Molecular Affinity by Biacore

The binding kinetics of the rLZ-8 to human EGFR was measured using the Biacore T200 (GE Healthcare). EGFR protein was immobilized onto a CM5 research grade sensor chip and rLZ-8*^wt^* or rLZ-8*^Mut 1-7^* were injected at concentrations ranging from 4 to 512 nM. On another sensor chip, rLZ-8 was immobilized onto chip and EGFR*^wt^* EGFR*^Mut 1^* or EGFR*^Mut 2^* were injected at concentrations ranging from 4 nM to 512 nM. Data acquisition for each concentration was followed by evaluation using the BIA Evaluations 3.2 program to determine the rate constants k_on_ and k_off_. K_D_ was determined according to the ratio of the rate constants k_off_/k_on_.

### 4.11. Relative Determination of EGFR Dimerization by In Situ PLA

PLA was conducted in Hep3B cells using the Duolink kit (Sigma-Aldrich, St. Louis, MO, USA). The samples were prepared and PLA was performed as described previously [47]. Cells were grown on coverslips and stimulated with 100 ng/mL EGF or 10 μg/mL rLZ-8 for 3 min. EGFR dimerization analysis was conducted using p-EGFR (Tyr1068) and p-EGFR (Tyr1173) primary antibodies. Finally, the samples were imaged using an OMX Imaging System.

### 4.12. Western Blot

Hep3B cells were cultured with rLZ-8, EGF or other reagents for the different time points. The cells were washed with ice-cold PBS and lysed in radio immunoprecipitation assay (RIPA) buffer containing 0.1% protease inhibitor cocktail. After centrifugation, the total protein concentration in the cell lysate was determined by bicinchoninic acid assay (BCA) according to the manufacturer’s instructions. The concentration of total proteins was determined using an automated capillary-based size sorting system (WES, ProteinSimple, Santa Clara, CA, USA). All the procedures were performed according to the manufacturer’s instructions. Five microliters of cell lysate and primary antibodies were used to detect the relative protein and the data were analyzed with an inbuilt Compass software (ProteinSimple).

### 4.13. Statistical Analysis

All statistical analyses were performed with GraphPad Prism 8 (GraphPad Software, San Diego, CA, USA). All data represent means ± SD. The animal experimental data were processed with a two-tailed Mann-Whitney test. The cellular experimental data were subjected to a nonparametric Kruskal-Wallis test to determine statistical significance. All experiments were performed at least in three biological replicates.

## 5. Conclusions

Taken together, we investigated a novel epitope on EGFR for HCC therapy. The epitope could cause catastrophic internalization of EGFR via macropinocytosis and recycling blockage of cell membrane. Thus, the novel oncotarget of EGFR in our studies may facilitate the design and development of more effective anti-EGFR agents for HCC clinical therapy. The related characteristic indexes of the structural domain provide an optimized direction for monoclonal antibodies and antibody-drug conjugates designing targeting EGFR.

## Figures and Tables

**Table 1:** Mutants of rLZ-8 and Epidermal Growth Factor Receptor (EGFR).

Mutations	Mutation Sites
rLZ-8*^Mut 1^*	K41A
rLZ-8*^Mut 2^*	K41D
rLZ-8*^Mut 3^*	K16A/S18A/K41A/D45A
rLZ-8*^Mut 4^*	K16A/K41A
rLZ-8*^Mut 5^*	L17K
rLZ-8*^Mut 6^*	D20H
rLZ-8*^Mut 7^*	D70K
EGFR*^Mut 1^*	S222A/K269A
EGFR*^Mut 2^*	S196A/S222A/K269A/S282A

## Supplementary Materials

The following are available online at https://www.mdpi.com/2072-6694/12/2/456/s1, Figure S1: rLZ-8 was recombinant expressed in Pichia pastoris, Figure S2: Transmission electron micrographs of Hep3B cells with rLZ-8 incubating, Figure S3: Impact of endocytic inhibitors on internalization of rLZ-8, Figure S4: rLZ-8 could be intensely internalized into cells by macropinocysosis, Figure S5: rLZ-8 stayed at late endosomes stage as no fusing with lysosomes, Figure S6: Rab7 activation and the effect of CaCl_2_ with rLZ-8 treating on Hep3B cells, Figure S7: rLZ-8 was co-localized with F-actin, Figure S8: Membrane ruffling and cell rounding induced by rLZ-8, Figure S9: Impact of rLZ-8 on internalization of BSA, Figure S10: rLZ-8 was not co-localization with other receptors, Figure S11: The tumor inhibitory rate of rLZ-8 was correlative with the EGFR expression in PDX models, Figure S12: IHC detection of tumor tissues of PDX models, Figure S13: IHC detection of rLZ-8 treating on mice, Figure S14: EGFR siRNA inhibited the internalization of rLZ-8, Figure S15: Mapping the rLZ-8/EGFR binding interface by CXMS, Figure S16: Biacore analysis of EGFR and rLZ-8, Figure S17: Binding of EGFR and rLZ-8 mutant on cell membrane, Figure S18: The effect of TriCEPS on the internalization of rLZ-8, Figure S19: Western blot files with molecular weight markers, Supplementary Material 1: Descriptions macropinocytosis characterization induced by rLZ-8, Supplementary Material 2: Descriptions of Rab7 activation and CaCl_2_ influence, Supplementary Material 3: Descriptions of immunohistochemistry and siRNA, Supplementary Material 4: Descriptions of binding analysis of rLZ-8 and EGFR, Video S1: Real-time imaging of Hep3B cell death induced by rLZ-8.
