# Supplementary material for: An Epitope on EGFR Loading Catastrophic Internalization Serve as a Novel Oncotarget for Hepatocellular Carcinoma Therapy
Source: Cancers (Basel). 2020 Feb 16;12(2):456. doi: 10.3390/cancers12020456 (PMC7072198; doi:10.3390/cancers12020456)
Supplement: Supplementary file 1 [file cancers-12-00456-s001.zip › cancers-710328-suppl-XML/cancers-710328-supp-figures-XML.pdf]

Supplementary Materials: An Epitope on EGFR Loading Catastrophic Internalization Serve as a Novel Oncotarget for Hepatocellular Carcinoma Therapy

Dianshuai Huang, Qingjie Fan, Zhiyi Liu, Shuqin Zhang, Wei Huang, Hongrui Li, Chongyang Liang and Fei Sun

Supplementary Material 1

The reorganization of F-actin is a major feature of macropinocytosis. As compared with the control, F-actin was not observed while treat with rLZ-8 in the cell abdomen suggesting that fragmented actin participated in the component of endosomes (Figure S4A). Macropinocytosis is a cholesterol-dependent process, and the rLZ-8 internalization efficiency was inhibited 50% using a cholesterol inhibitor, mβCD (Figure S4B). Since GTPases regulated the macropinocytosis process, we evaluated their roles in rLZ-8 internalization. The results illustrated that Ras GTP, Arf6 GTP, and Rac1 GTP were all activated in the internalization of rLZ-8, which was not similar to the internalization of BSA (Figure S4C). BSA-mediated activation returned to normal level 1 h after internalization, whereas rLZ-8 still remained in the high activity status.

Supplementary Material 2

As illustrated in Figure S6A, the internalization of rLZ-8 led to a relatively low level of Rab7 GTP consumption. By contrast, Rab7 consumption was increased during the internalization of BSA, indicating that LE containing BSA fused with lysosomes.

Luzio et al. reported that regulation of Ca^{2+} release inhibited the fusion of late endosomes (LE) with lysosomes, and the addition of sufficient $CaCl_2$ recovered the fusion. In our study, the addition of $CaCl_2$ failed to induce fusion between LE containing rLZ-8 and lysosomes in the Figure S6B.

Supplementary Material 3

In immunohistochemistry assay, we selected mouse HCC tumors which examined in Figure 1C. The staining intensity of rLZ-8 increased in a dose-dependent manner (Figure S11), and displayed similar subcellular localization with EGFR.

We inhibited the expression of EGFR and the related kinases, K-Ras and c-Src. Figure S12 showed that the low expression level of EGFR on the cell surface inhibited the internalization of rLZ-8. Inhibition of c-Src and K-Ras significantly decreased the amount of rLZ-8 entering into cells; however, it was not higher than during the absence of EGFR expression.

Supplementary Material 4

The binding analysis of rLZ-8$^{Mut\ 1,2,4}$ and EGFR on cell membrane showed a decrease in the binding rate than rLZ-8^{wt} (Figure S15). Another confirmatory result is that we conjugated rLZ-8 and TriCEPS (a small molecule specifically binds to lysine residue in the protein), the rate of internalization of the complex in Hep3B is less than that of rLZ-8 significantly (Figure S16).

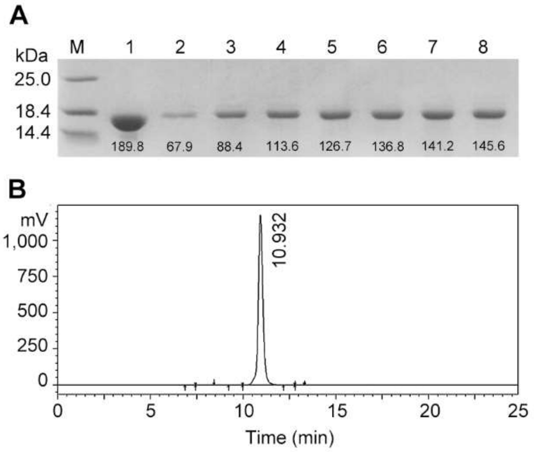

Figure S1. rLZ-8 was recombinant expressed in *Pichia pastoris*. (**A**) The *Pichia pastoris* X33 transformants with rLZ-8 gene were cultured in BioFlo 310 Bioreactor as shown in Materials and Methods. Samples were taken on different times and rLZ-8 expression was detected by SDS-PAGE. Lane 1: Quantitative standard of rLZ-8; lane 2–8: samples with 60–96 h culturing (one sample every 6 h). (**B**) rLZ-8 was purified by a Superdex TM-75 prep grade column and the purity of rLZ-8 was analysed by HPLC.

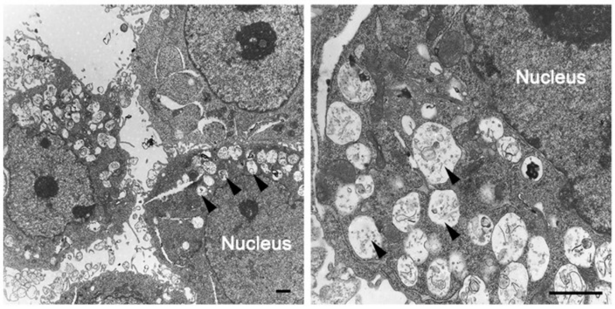

Figure S2. Transmission electron micrographs of Hep3B cells with rLZ-8 incubating. Arrowheads show ring-like vesicles. Scale Bars on left, 5 μm, on right, 1 μm.

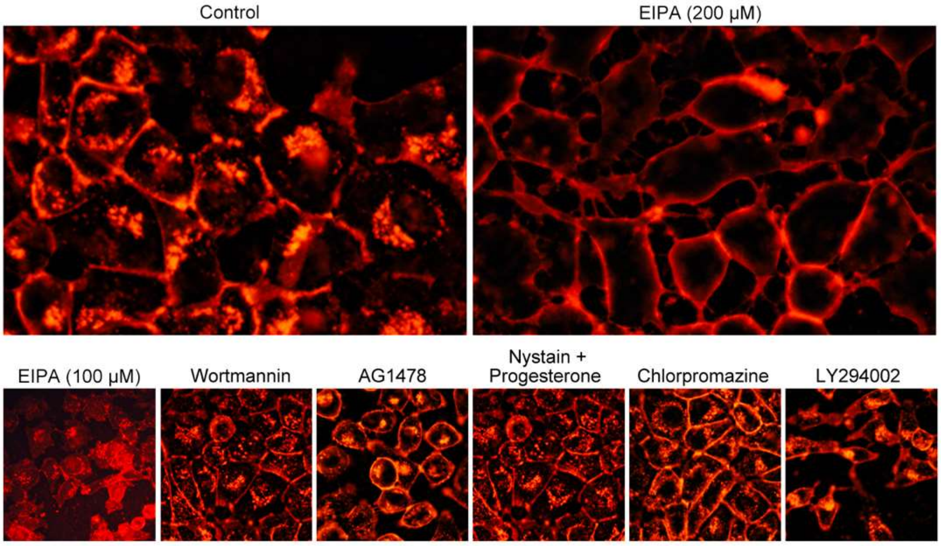

Figure S3. Impact of endocytic inhibitors on internalization of rLZ-8. 5 μg/mL rLZ-8 (red) treated on Hep3B cells for 2 h after different inhibitors pre-incubated for 30 min or not. Then cells were imaged by confocal microscopy. Magnification ×200.

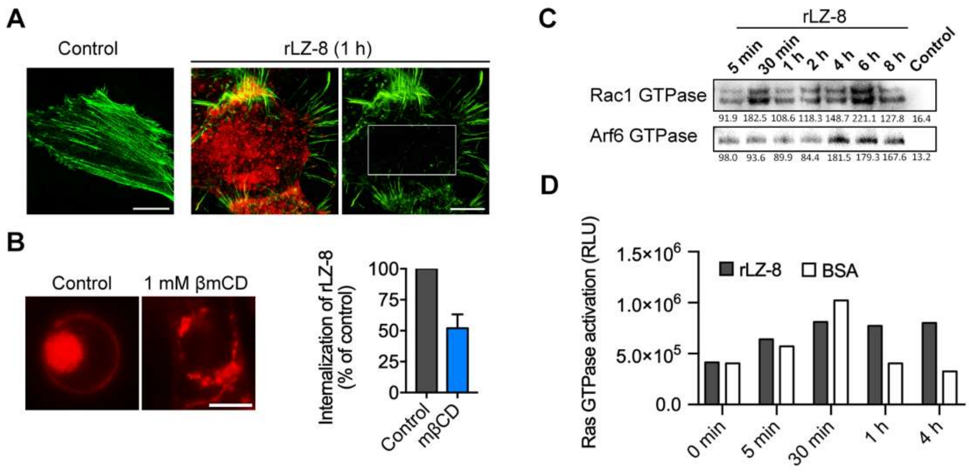

Figure S4. rLZ-8 could be intensely internalized into cells by macropinocysosis. (**A**) Actin (green) stained by phalloidin 488 with or without 10 μg/mL rLZ-8 (red) treating for 1 h. The square showed disappearance of F-actin with string structure. Bars, 10 μm. (**B**) 5 μg/mL rLZ-8 (red) treated on Hep3B cells for 2 h after 1 mM mβCD pre-incubated for 30 min or not. Then cells were imaged. Internalization of rLZ-8 (% of control) was analysed by Imaris software. All data are means ± SD. Scale Bars, 10 μm. (**C**) Detection of Rac1 GTPase and Arf6 GTPase activation by relative kits. 10 μg/mL rLZ-8 treated on Hep3B cells before cells lysing. Rac1 GTPase and Arf6 GTPase isolated as kits protocols, then detected by western blotting. (D) Detection of Ras GTPase activation by Ras Activation ELISA Assay Kit. 10 μg/mL rLZ-8 or 100 μg/mL BSA treated on Hep3B cells before ELISA detection.

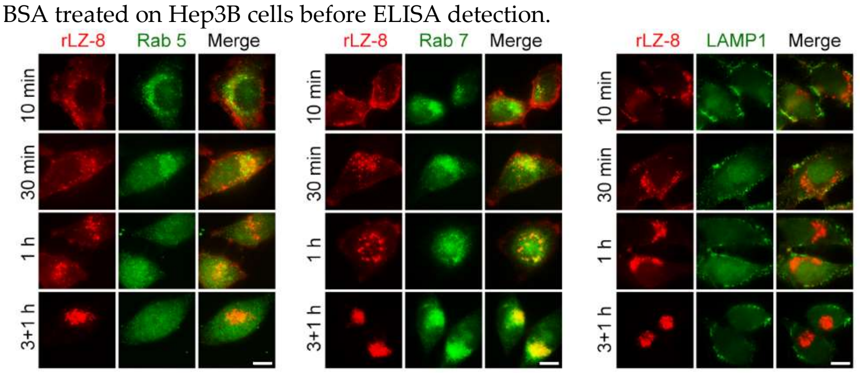

Figure S5. rLZ-8 stayed at late endosomes stage as no fusing with lysosomes. Immunofluorescent staining of Rab5, Rab7 or LAMP1 (green) after 10 μg/mL rLZ-8 (red) treated for different time. For 3 + 1 h group, rLZ-8 was removed after treatment for 3 h and cells incubation lasted 1 h. Scale Bars, 10 μm.

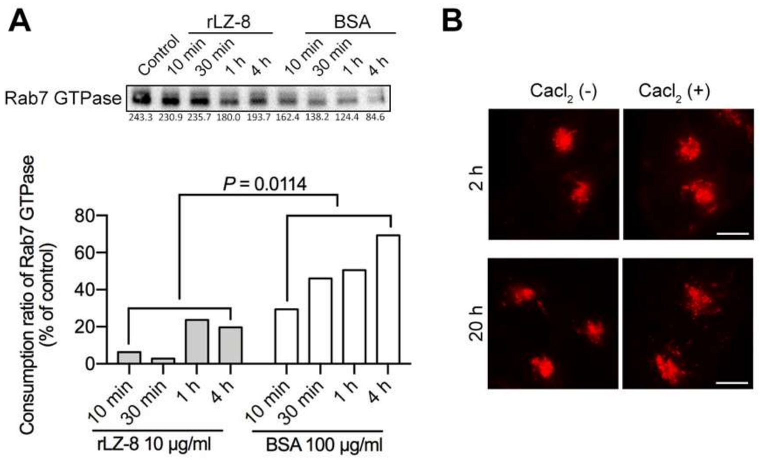

Figure S6. Rab7 activation and the effect of $CaCl_2$ with rLZ-8 treating on Hep3B cells. (**A**) Determination of the activation status of Rab7. Lysates were prepared after 20 µg/mL rLZ-8 or 100 µg/mL BSA incubation. Active Rab7 was quantified by pull-down assay. The decrease of Rab7 GTPase activation (% of control) was analysed by Imaris software. Statistical significance was calculated with a nonparametric Kruskal-Wallis test, in which the *P*-value was shown in panel. (**B**) 10 µg/mL rLZ-8 (red) treated on Hep3B cells for 4 h and then be removed. Then 0.5 µM $CaCl_2$ treated on cells for 2 h/20 h or not before imaging. Scale Bars, 10 µm.

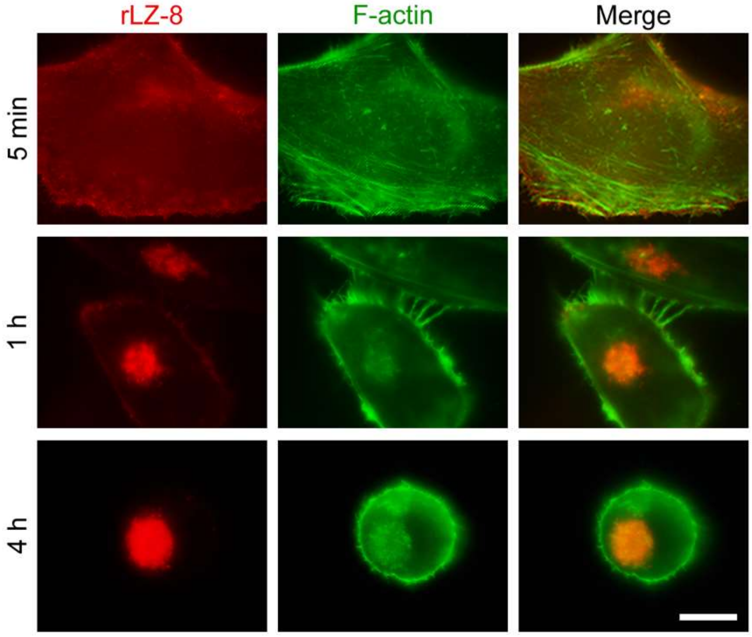

Figure S7. rLZ-8 was co-localized with F-actin. F-actin (green) stained by ActinGreenTM 488 ReadyProbes with 10 µg/mL rLZ-8 (red) treating on Hep3B cells. Scale Bars, 10 µm.

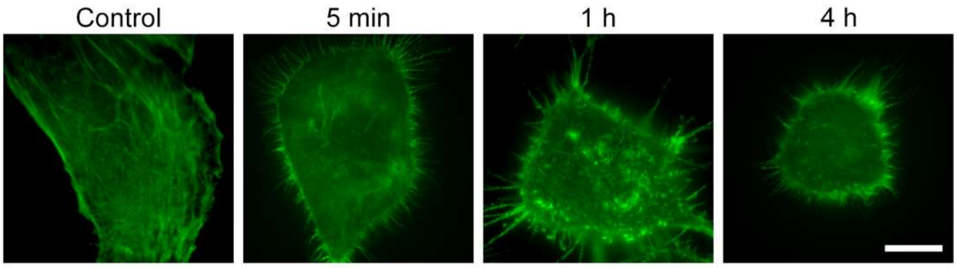

Figure S8. Membrane ruffling and cell rounding induced by rLZ-8. F-actin (green) stained by ActinGreenTM 488 ReadyProbes with 10 μg/mL rLZ-8 treating on Hep3B cells. Cells were imaged after different treating time of rLZ-8. Scale Bars, 10 μm.

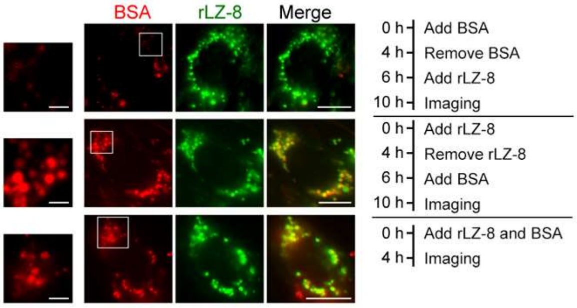

Figure S9. Impact of rLZ-8 on internalization of BSA. 1 μg/mL rLZ-8 (green) and 100 μg/mL BSA (red) treatment of Hep3B cells at varying levels are shown beside the panel. The square shows the differential status of BSA endosomes under different doses of rLZ-8. Scale Bars in left, 1 μm, in right, 10 μm.

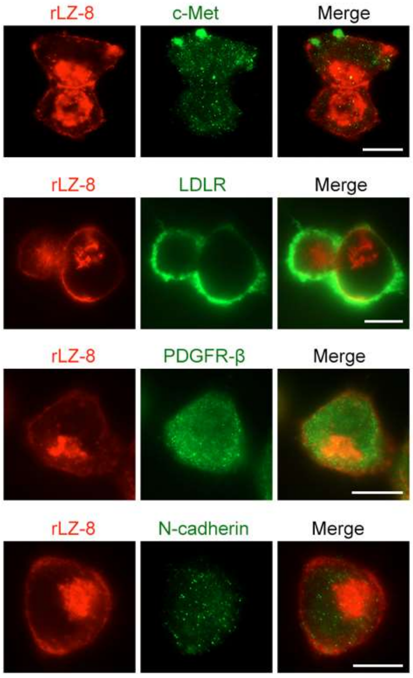

Figure S10. rLZ-8 was not co-localization with other receptors. Immunofluorescent staining of c-Met, LDLR, PDGFR-β and N-cadherin (green) following exposure of Hep3B cells to 10 μg/mL rLZ-8 (red) for 3 h. Scale Bars, 10 μm.

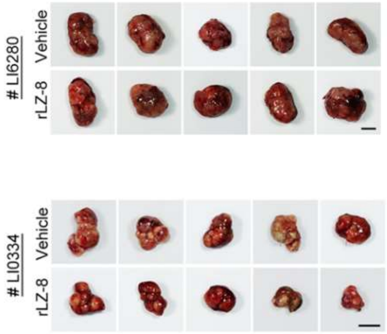

Figure S11. The tumor inhibitory rate of rLZ-8 was correlative with the EGFR expression in PDX models. Different types of PDX models (LI6280, LI1097, LI0050, LI0334, LI6611) of HCC were established as shown in Methods. Mice were divided into groups and dosed with normal saline control or 5 mg/kg rLZ-8. All mice were injected once daily for total 28 days. Tumors were dissected at 28 d post inoculation and imaged. Scale Bars, 1 cm.

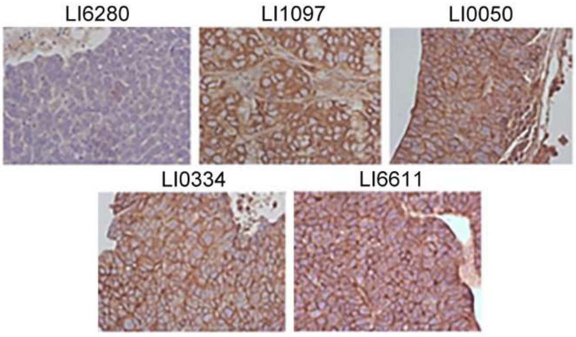

Figure S12. IHC detection of tumor tissues of PDX models. IHC detection in different tumor tissues of PDX models (LI6280, LI1097, LI0050, LI0334, LI6611). EGFR antibody was used for staining. Magnification ×200.

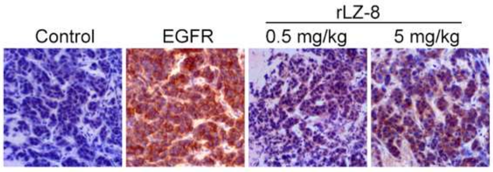

Figure S13. IHC detection of rLZ-8 treating on mice. IHC detection in selected mouse HCC tumors also examined in Figure 1C. rLZ-8 and EGFR antibodies were used for staining, as anti-mouse IgG controlled. Magnification ×200.

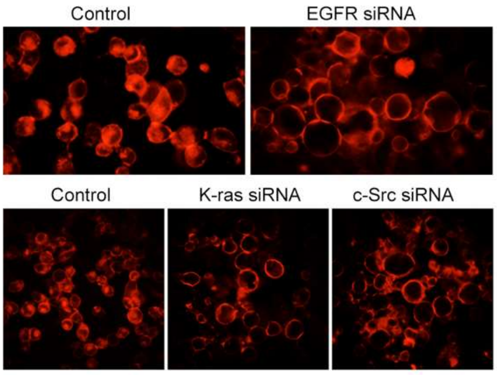

Figure S14. EGFR siRNA inhibited the internalization of rLZ-8. 100 nM EGFR siRNA, 200 nM K-Ras or c-Src siRNA treated on Hep3B cells for 6 h together with Lipofectamine 2000 and then be removed. 10 μg/mL rLZ-8 (red) treated on Hep3B cells for 2 h before confocal microscopy imaging. For control group, cells were only treated with Lipofectamine 2000. Magnification ×200.

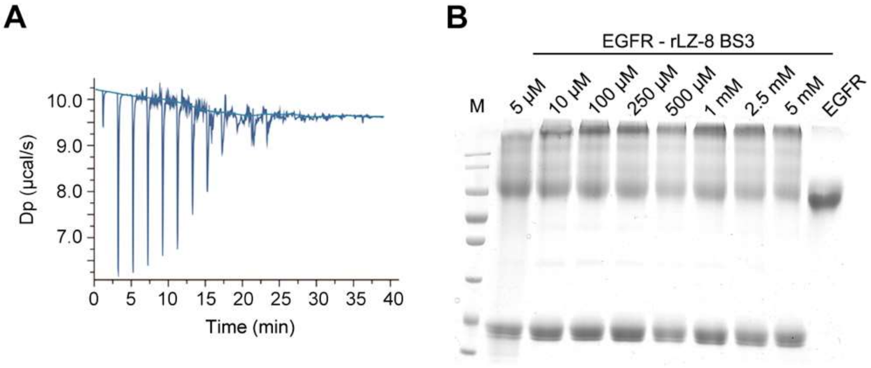

Figure S15. Mapping the rLZ-8/EGFR binding interface by CXMS. (**A**) ITC experiments employing purified EGFR ectodomain and rLZ-8. (**B**) The cross-linked EGFR/rLZ-8 complexes with DSS/BS3 were isolated by SDS-PAGE. The concentrations of EGFR-rLZ-8 were shown on lanes.

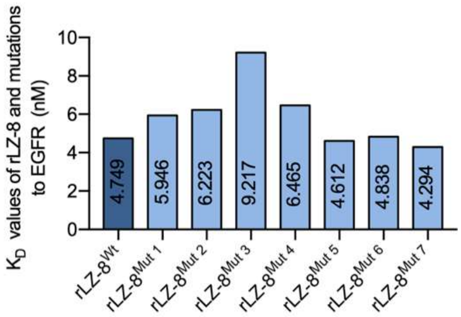

Figure S16. Biacore analysis of EGFR and rLZ-8. Recombinant human EGFR was immobilized onto sensor chips and rLZ-8*wt* or rLZ-8*Mut 1-7* were injected at concentrations ranging from 4 nM to 512 nM. K_D values were shown on panels.

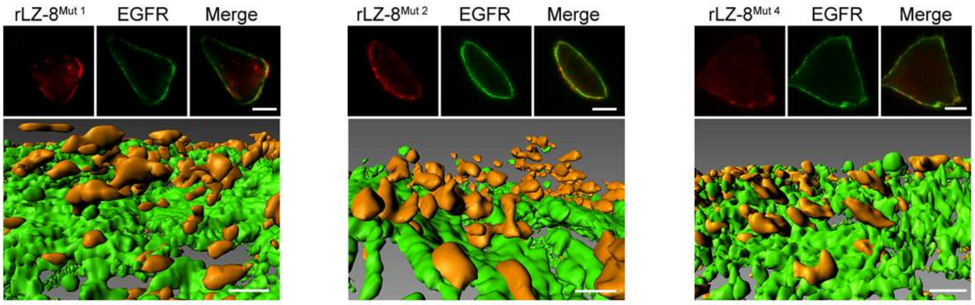

Figures S17. Binding of EGFR and rLZ-8 mutant on cell membrane. Hep3B cells were treated with 10 µg/mL rLZ-8*wt* or rLZ-8*Mut 1,2,4* (red) for 20 min, with EGFR (green) coloring by immunofluorescence (no permeabilized). Surface rendering was generated using Imaris software correlating rLZ-8*wt*/rLZ-8*Mut 1,2,4* and EGFR. Scale Bars above, 10 µm, bottom, 2 µm.

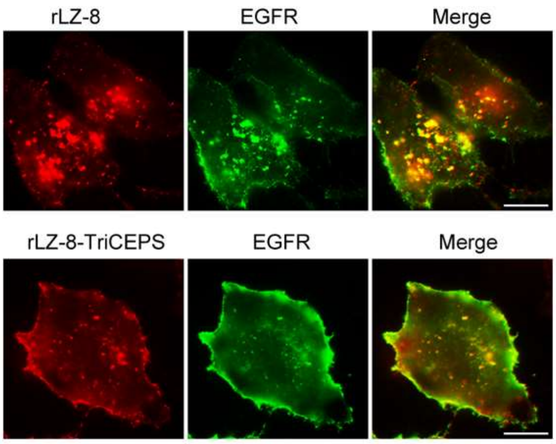

Figure S18. The effect of TriCEPS on the internalization of rLZ-8. TriCEPS is a small molecule from Dualsystems Biotech (Switzerland) which could bind with proteins by attaching to lysine. rLZ-8-TriCEPS 10 μg/mL rLZ-8 or rLZ-8-TriCEPS (red) treated on Hep3B cells for 1 h. Immunofluorescent stained of EGFR (green) before imaging. Scale Bars, 10 μm.

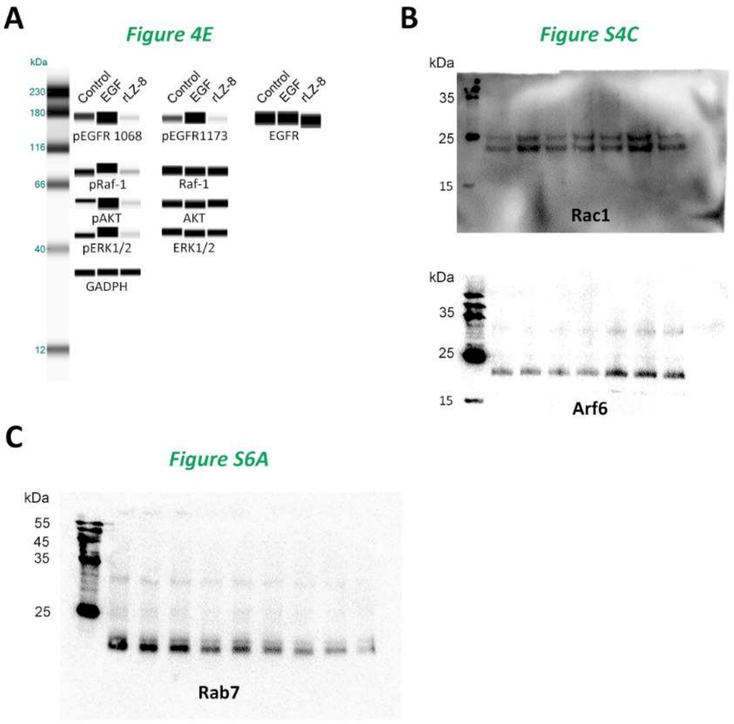

Figure S19. Western blot files with molecular weight markers. (**A**) The expression of relative proteins of EGFR pathways was detected using WES system. (**B**) Detection of Rac1 GTPase and Arf6 GTPase activation by relative kits. (**C**) Determination of the activation status of Rab7 by western blot analysis.

© 2020 by the authors. Licensee MDPI, Basel, Switzerland. This article is an open access article distributed under the terms and conditions of the Creative Commons Attribution (CC BY) license (http://creativecommons.org/licenses/by/4.0/).
